# Comorbidities of obesity in school children: a cross-sectional study in the PIAMA birth cohort

**DOI:** 10.1186/1471-2458-10-184

**Published:** 2010-04-09

**Authors:** Alet H Wijga, Salome Scholtens, Wanda JE Bemelmans, Johan C de Jongste, Marjan Kerkhof, Maarten Schipper, Elisabeth A Sanders, Jorrit Gerritsen, Bert Brunekreef, Henriette A Smit

**Affiliations:** 1 Centre for Prevention and Health Services Research, National Institute for Public Health and the Environment, Bilthoven, The Netherlands; 2 Institute for Risk Assessment Sciences, Utrecht University, Utrecht, The Netherlands; 3 Department of Pediatrics, Division of Respiratory Medicine, Erasmus MC - Sophia, Rotterdam, The Netherlands; 4 Department of Epidemiology and Bioinformatics, University of Groningen, Groningen, the Netherlands; 5 Expertise Centre for Methodology and Information Services, National Institute for Public Health and the Environment, Bilthoven, The Netherlands; 6 Department of Pediatric Immunology & Infectious Diseases, Wilhelmina Children's Hospital, University Medical Centre Utrecht, Utrecht, The Netherlands; 7 Beatrix Children's Hospital, University Medical Centre, University of Groningen, Groningen, The Netherlands; 8 Julius Centre for Health Sciences and Primary Care, University Medical Centre Utrecht, Utrecht, The Netherlands

## Abstract

**Background:**

There is ample evidence that childhood overweight is associated with increased risk of chronic disease in adulthood. The aim of this study was to investigate associations between childhood overweight and common childhood health problems.

**Methods:**

Data were used from a general population sample of 3960 8-year-old children, participating in the Dutch PIAMA birth cohort study. Weight and height, measured by the investigators, were used to define BMI status (thinness, normal weight, moderate overweight, obesity). BMI status was studied cross-sectionally in relation to the following parental reported outcomes: a general health index, GP visits, school absenteeism due to illness, health-related functional limitations, doctor diagnosed respiratory infections and use of antibiotics.

**Results:**

Obesity was significantly associated with a lower general health score, more GP visits, more school absenteeism and more health-related limitations, (adjusted odds ratios around 2.0 for most outcomes). Obesity was also significantly associated with bronchitis (adjusted odds ratio (aOR) and 95% confidence intervals (95%CI): 5.29 (2.58;10.85) and with the use of antibiotics (aOR (95%CI): 1.79 (1.09;2.93)). Associations with flu/serious cold, ear infection and throat infection were positive, but not statistically significant. Moderate overweight was not significantly associated with the health outcomes studied.

**Conclusion:**

Childhood obesity is not merely a risk factor for disease in adulthood, but obese children may experience more illness and health related problems already in childhood. The high prevalence of the outcomes studied implies a high burden of disease in terms of absolute numbers of sick children.

## Background

Childhood overweight is becoming increasingly prevalent all over the world and this trend is seen as an alarming development by public health professionals. The most frequently stated reason for their concern is that overweight children tend to become overweight adults and that adult overweight is a strong risk factor for a number of chronic diseases [1]. Literature reviews show that most studies on the consequences of childhood overweight have indeed focused on early symptoms or risk factors for later chronic diseases such as diabetes and cardiovascular disease [2-5]. For overweight children and their parents, current overweight-related health problems, might be more reason for concern than the risk of adverse health effects in future. The objective of this study was to investigate the general health status of overweight children and the possible impact of overweight on diseases that have a high prevalence in childhood.

In a general population sample of 3,960 8-year-old children, we compared overweight to normal weight children with respect to the following indicators of general health: the RAND general health index, GP (general practitioner) visits, school absenteeism due to illness and health-related functional limitations. In addition, we studied respiratory infections, which are highly prevalent childhood illnesses, and the use of antibiotics in relation to overweight. The health outcomes studied, were reported by the parents in questionnaires and we hypothesized that these reports might be influenced by the parents' perception of their child's body shape. We therefore also investigated the role of parental perception in the associations between overweight and health.

## Methods

### Study design and study population

The study population consisted of children who participated in the Dutch Prevention and Incidence of Asthma and Mite Allergy (PIAMA) birth cohort study. In this study, pregnant women were recruited from the general population in three different regions of the Netherlands (n = 4,146). Their children were born in 1996-1997 and were followed up to the age of 8 years. A detailed description of the study design has been published previously [6].

Data were collected mainly by annual postal questionnaires, which included questions on the child's weight and height, lifestyle and different aspects of health and disease. At the age of 8 years, the children were invited for a medical examination, which included measurement of weight and height. The study protocol was approved by the medical ethics committees of the participating institutes and all parents gave written informed consent.

Of the baseline population of 4,146 mothers, 186 (5%) were lost to follow-up before any data on the child had been collected. The study therefore started with 3,960 newborns. When the medical examination of 8-year-olds was planned, 3,668 children (88% of 4,146) were still in the study, 3,522 children were invited and 2,214 participated (63% of those invited). When the questionnaires on 8-year-olds were sent out, 3,653 families were still in the study and received the questionnaire. Questionnaires were completed by 3,268 families (90% of those who received the questionnaire and 79% of the baseline study population).

### Assessment of exposure: BMI status

The 8-year-old children were weighed and measured in their underwear by trained research staff using calibrated measuring equipment. Weight was measured to 0.1 kg and height to 0.1 cm. BMI (body mass index: weight (kg)/height (m)^2^) was calculated and 'moderate overweight' and 'obesity' were defined according to age and gender specific international standards that use cut-off points equivalent to the 25 kg/m^2 ^and 30 kg/m^2 ^cut-offs that are commonly used for adults [7]. We use the term 'overweight' for the total group of children who are either moderately overweight or obese. Because thinness too may be associated with a child's health, we considered thin children as a separate group apart from normal weight and overweight children. Thinness was defined using international age and gender specific cut-off points equivalent to an adult BMI of 18.5 kg/m^2 ^[8].

### Assessment of health outcomes

Data from the questionnaires filled in by the parents when the children were 8 years old, were used to investigate the following health outcomes as indicators of general health, health related problems and (respiratory) infections:

- The RAND general health rating index for children, which is based on 7 questions about the child's general health and susceptibility to illness. The total score has a minimum of 7 and a maximum of 32 and a higher score indicates a better health [9,10].

- School absenteeism due to illness (at least 1 day in the last 2 months); health related limitations (a positive answer to the question "Did your child face any health-related limitations in daily functioning during the last 2 months?"); GP contacts (at least 1 in the last 2 months) (Note: in the Netherlands, general practitioners and not pediatricians provide primary care for children).

- Doctor diagnosed flu/serious cold, doctor diagnosed throat infection, doctor diagnosed ear infection and doctor diagnosed bronchitis in the last 12 months and use of antibiotics in the last 12 months.

### Data analyses

Cross-sectional associations between BMI status and the outcome variables were analysed by linear regression for continuous outcomes and by logistic regression for dichotomous outcomes.

The regression models were adjusted for gender, maternal education (low, intermediate, high), breast feeding (never, 0-16 weeks or > 16 weeks), region (North, West, central) and smoking in the home at least once a week (yes/no). Data on confounders were obtained from the questionnaire that was administered at 1 year, except tobacco smoke exposure which was obtained from the questionnaire at 8 years. Apart from the confounders mentioned above, we also assessed the role of parental perception of the child's body shape, using the answers to the following 2 questions from the questionnaire for 8-year-olds: "Do you find your child rather heavy or chubby? (yes/no)"; and "Do you find your child rather slim or slender? (yes/no)".

For 3,120 children, complete questionnaire data on each of the health outcomes were available. Weight and height were measured in 2,214 children. Of the original 3,960 newborns in the PIAMA study, 1,997 children had complete data on health outcomes as well as measured weight and height at 8 years of age and 1,971 of these children also had complete data on confounders. Of all the potentially available data (15 variables × 3960 subjects = 59400 data) 15% was missing, but 50% of the study population had missing data on at least 1 of the variables and 44% had missing data on BMI. With half of the baseline population having incomplete data, a complete case analysis does not make efficient use of the data, because a large amount of the available information is discarded, resulting in imprecise estimates of associations. In addition, if data are not 'missing completely at random' (MCAR), complete case analysis may lead to biased results [11,12]. If the probability of data being missing is associated with variables measured in the study and is not influenced by unobserved external factors, multiple imputation is an appropriate method to avoid bias that may occur in complete case analysis due to selective missing of data. In our dataset, the probability of data being missing was strongly associated with factors that were measured in the study. Although the possibility that also unmeasured factors may have influenced data being missing, can never be excluded in observational studies, we therefore chose multiple imputation as the best available method to deal with missing data in our study.

Missing data were multiple times imputed, using the 'Multivariate Imputation by Chained Equations' (MICE) procedure [13,14], that runs under the statistical program R version 2.5.0 [15]. The variables used to impute the missing BMI status measurements at the age of 8 years included all data used in the analyses and, in addition, data on BMI status at the ages 0-8 years based on annual parental reports of the child's weight and height. After 100 iterations, convergence was achieved resulting in 20 imputed datasets. Each imputed dataset was analyzed by standard complete data procedures, which ignore the distinction between real and imputed values. The results of the analyses were combined using PROC MIANALYZE in SAS. All analyses were performed on the complete case data and on the imputed data. In the paper we present the results of the analyses in the imputed data. In addition, the main results of the analyses in the complete cases are presented for comparison.

## Results

### Characteristics of the study population

Table 1 shows the characteristics of the study population for children with complete data on the health outcomes, for the children with complete data on both the exposure and the outcome variables, and for the imputed data set.

**Table 1:** Characteristics (in percentages, unless indicated otherwise) of the study population

	Children with complete data on BMI status and health outcomes	Children with complete data on the health outcomes	Imputed data set n = 3960
Gender	n = 1997	n = 3120	
boys	50.3	51.3	51.7

Birth weight (grams)	n = 1991	n = 3108	
mean (s.d.)	3522 (537)	3524 (535)	3507 (546)

Region	n = 1997	n = 3120	
North	31.2	31.4	31.1
Central	42.1	41.7	40.0
West	26.7	26.9	28.9

Maternal education	n = 1991	n = 3106	
Low	19.8	21.3	23.8
Intermediate	42.1	42.0	41.4
High	38.1	36.7	34.7

Breast feeding	n = 1983	n = 3090	
Never	15.4	16.4	17.9
0-16 weeks	44.4	46.5	49.7
> 16 weeks	40.2	37.1	32.4

Smoking in the home at 1 year	n = 1992 25.3	n = 3110 28.0	26.0
Smoking in the home at 8 years	n = 1990 15.8	n = 3109 16.6	18.1

BMI status at 8 yrs	n = 1997	n = 1997	
Thinness	7.0	6.9	8.3
Normal weight	78.7	78.8	75.7
Moderate overweight	11.7	11.7	12.1
Obesity	2.7	2.7	3.9

RAND score	n = 1997	n = 3120	
mean (s.d.)	27.8 (3.5)	27.8 (3.5)	27.6 (3.6)

GP contact during last 2 months (yes)	n = 1997	n = 3120	
	20.7	20.6	22.1

Not to school due to illness in last 2 months (≥ 1 day)	n = 1997 27.8	n = 3120 28.1	29.4

Health-related limitations last 2 months (yes)	n = 1997 16.3	n = 3120 16.3	17.4

Flu/serious cold last 12 months (yes)	n = 1997 13.3	n = 3120 13.4	15.2

Throat infection last 12 months (yes)	n = 1997 4.9	n = 3120 4.7	5.7

Ear infection last 12 months (yes)	n = 1997 9.1	n = 3120 8.6	9.5

Bronchitis last 12 months (yes)	n = 1997 2.7	n = 3120 2.6	3.5

Antibiotics in last 12 months (yes)	n = 1997 15.8	n = 3120 15.7	17.2

In the group of children with missing data at the age of 8 years (data not shown), there was a relatively high prevalence of low maternal education (31.3%), of no breast feeding (20.2%) and of smoking in the home at the age of 1 year (30.8%). As expected, in the imputed data the prevalence of these characteristics was somewhat higher than in the children with complete data. In the imputed data also the prevalence of thinness and moderate overweight were slightly higher than in the complete cases and the prevalence of obesity was 3.9% in the imputed data set as compared to 2.7% in the group of children with complete data (table 1). The prevalence of the health outcomes in the imputed data was also slightly higher than the prevalence in the complete cases.

### Associations between BMI status and health

Tables 2 and 3 show the prevalence of the health outcomes by BMI status and the associations of thinness, moderate overweight and obesity (with normal weight as the reference) with the health outcomes, for the complete cases and the imputed data respectively. The data consistently show poorer health in obese children for each of the 4 general health outcomes. Obesity was associated with a lower RAND score, with increased likelihood of GP visit, health-related limitations and school absenteeism (adjusted odds ratios around 2.0, see table 3). The data suggest that these problems could partly result from a higher incidence of respiratory infections. The associations between obesity and the respiratory infections were positive, and the association with bronchitis was statistically significant, as was the association between obesity and the use of antibiotics (table 3). For most health outcomes, the results seem suggestive of a dose-response relationship, with increasing risk from moderate overweight to obesity. The associations between moderate overweight and the health outcomes were mostly weak however and not statistically significant. Thinness showed a statistically significant association with a lower RAND score, but not with any of the other health outcomes.

**Table 2:** BMI status and health outcomes: Means and prevalence of health outcomes by BMI status; crude and adjusted^1 ^odds ratios (for dichotomous variables) and regression coefficients (for continuous variables) with 95% confidence intervals; complete cases n = 1971

	BMI status			
**Health outcome**	**Thinness** **n = 138**	**Normal weight** **n = 1555**	**Moderate overweight** **n = 227**	**Obesity** **n = 51**

RAND score				
Mean (s.d.)	26.9 (3.5)	28.0 (3.4)	27.3 (3.5)	26.5 (3.9)
β (95% CI)	-1.04 (-1.65; -0.44)		-0.64 (-1.12; -0.16)	-1.51 (-2.47; -0.54)
adj. β (95% CI)	-1.02 (-1.62; -0.42)		-0.62 (-1.10; -0.13)	-1.45 (-2.41; -0.48)

GP contact during last 2 months (yes)				
%	24.5	20.1	18.9	33.3
OR (95% CI)	1.28 (0.86;1.93)	1.00	0.93 (0.65;1.32)	1.98 (1.09;3.60)
aOR (95% CI)	1.26 (0.84;1.90)	1.00	0.91 (0.64;1.30)	1.96 (1.08;3.57)

Not to school due to illness in last 2 months (≥ 1 day)				
%	28.8	26.8	33.0	35.3
OR (95% CI)	1.11 (0.75;1.62)	1.00	1.35 (1.00;1.82)	1.49 (0.83;2.68)
aOR (95% CI)	1.12 (0.76;1.65)	1.00	1.36 (1.00;1.83)	1.53 (0.85;2.75)

Health-related limitations last 2 months (yes)				
%	20.1	15.4	17.6	29.4
OR (95% CI)	1.38 (0.89;2.14)	1.00	1.17 (0.81;1.69)	2.28 (1.23;4.23)
aOR (95% CI)	1.36 (0.88;2.11)	1.00	1.21 (0.84;1.76)	2.31 (1.24;4.31)

Flu/serious cold last 12 months (yes)				
%	11.5	11.8	22.9	23.5
OR (95% CI)	0.98 (0.57;1.68)	1.00	2.23 (1.58;3.15)	2.31 (1.19;4.48)
aOR (95% CI)	0.95 (0.55;1.64)	1.00	2.22 (1.57;3.16)	2.27 (1.16;4.43)

Throat infection last 12 months (yes)				
%	4.3	4.8	6.2	5.9
OR (95% CI)	0.89 (0.38;2.08)	1.00	1.30 (0.72;2.34)	1.23 (0.38;4.05)
aOR (95% CI)	0.90 (0.38;2.10)	1.00	1.23 (0.68;2.23)	1.19 (0.36;3.93)

Ear infection last 12 months (yes)				
%	10.8	8.8	9.3	13.7
OR (95% CI)	1.26 (0.72;2.22)	1.00	1.06 (0.66;1.72)	1.66 (0.73;3.75)
aOR (95% CI)	1.24 (0.71;2.19)	1.00	1.06 (0.65;1.72)	1.67 (0.74;3.81)

Bronchitis last 12 months (yes)				
%	4.3	2.3	2.2	11.8
OR (95% CI)	1.96 (0.81;4.74)	1.00	0.98 (0.38;2.52)	5.79 (2.32;14.45)
aOR (95% CI)	2.05 (0.84;5.00)	1.00	0.99 (0.38;2.57)	5.70 (2.25;14.44)

Antibiotics in last 12 months (yes)				
%	15.8	15.3	18.1	23.5
OR (95% CI)	1.05 (0.65;1.68)	1.00	1.23 (0.85;1.77)	1.71 (0.88;3.31)
aOR (95% CI)	1.02 (0.63;1.65)	1.00	1.17 (0.81;1.70)	1.64 (0.84;3.20)

^1 ^Adjusted for gender, region, maternal education, breast feeding and smoking in the home at age 8

**Table 3:** BMI status and health outcomes: Means and prevalence of health outcomes by BMI status; crude and adjusted^1 ^odds ratios (for dichotomous variables) and regression coefficients (for continuous variables) with 95% confidence intervals; imputed data n = 3960

	BMI status			
**Health outcome**	**Thinness** **n = 327**	**Normal weight** **n = 2999**	**Moderate overweight** **n = 480**	**Obesity** **n = 154**

RAND score				
Mean (s.d.)	26.9 (3.8)	27.8 (3.5)	27.2 (3.7)	26.4 (4.1)
β (95% CI)	-0.98 (-1.52; -0.44)		-0.64 (-1.03; -0.25)	-1.42 (-2.24; -0.61)
adj. β (95% CI)	-0.94 (-1.49; -0.39)		-0.59 (-0.98; -0.20)	-1.29 (-2.10; -0.47)

GP contact during last 2 months (yes)				
%	25.4	21.1	21.1	37.9
OR (95% CI)	1.28 (0.93;1.74)	1.00	1.00 (0.75;1.34)	2.28 (1.47;3.56)
aOR (95% CI)	1.24 (0.90;1.71)	1.00	0.98 (0.73;1.30)	2.17 (1.38;3.42)

Not to school due to illness in last 2 months (≥ 1 day)				
%	31.9	28.0	33.1	41.1
OR (95% CI)	1.20 (0.88;1.64)	1.00	1.27 (0.96;1.67)	1.79 (1.17;2.73)
aOR (95% CI)	1.22 (0.89;1.67)	1.00	1.28 (0.97;1.68)	1.82 (1.19;2.78)

Health-related limitations last 2 months (yes)				
%	19.8	16.4	18.9	28.5
OR (95% CI)	1.26 (0.88;1.80)	1.00	1.19 (0.90;1.57)	2.02 (1.24;3.30)
aOR (95% CI)	1.24 (0.86;1.78)	1.00	1.19 (0.91;1.57)	1.98 (1.21;3.25)

Flu/serious cold last 12 months (yes)				
%	15.8	13.7	22.9	20.7
OR (95% CI)	1.18 (0.79;1.76)	1.00	1.87 (1.39;2.53)	1.63 (0.94;2.81)
aOR (95% CI)	1.13 (0.76;1.69)	1.00	1.83 (1.35;2.48)	1.50 (0.86;2.63)

Throat infection last 12 months (yes)				
%	5.5	5.4	7.1	8.1
OR (95% CI)	1.02 (0.55;1.88)	1.00	1.34 (0.86;2.09)	1.45 (0.51;4.11)
aOR (95% CI)	1.02 (0.55;1.89)	1.00	1.24 (0.79;1.93)	1.34 (0.46;3.88)

Ear infection last 12 months (yes)				
%	10.6	9.1	9.4	15.5
OR (95% CI)	1.17 (0.67;2.02)	1.00	1.03 (0.69;1.54)	1.80 (0.87;3.73)
aOR (95% CI)	1.12 (0.65;1.95)	1.00	0.98 (0.66;1.47)	1.64 (0.79;3.39)

Bronchitis last 12 months (yes)				
%	5.7	2.8	2.9	13.8
OR (95% CI)	2.04 (0.86;4.85)	1.00	1.01 (0.49;2.09)	5.52 (2.75;11.08)
aOR (95% CI)	2.07 (0.88;4.88)	1.00	0.98 (0.48;2.02)	5.29 (2.58;10.85)

Antibiotics in last 12 months (yes)				
%	17.8	16.1	20.6	27.5
OR (95% CI)	1.12 (0.74;1.70)	1.00	1.35 (1.00;1.83)	1.96 (1.19;3.22)
aOR (95% CI)	1.09 (0.71;1.66)	1.00	1.27 (0.94;1.72)	1.79 (1.09;2.93)

^1 ^Adjusted for gender, region, maternal education, breast feeding and smoking in the home at age 8

### The role of respiratory infections

Our results show that the obese children had a lower mean RAND score and a higher prevalence of GP visits, school absenteeism due to illness and health related limitations than the normal weight children. The data suggest that these health problems could partly result from a higher incidence of respiratory infections. To investigate this issue in more detail, we analysed the associations between BMI status and these health outcomes in the sub-group of children who did not have doctor diagnosed flu/serious cold, ear infection, throat infection or bronchitis in the last 12 months. In this sub-group (about 75% of all children) the mean RAND score was higher and the prevalence of GP visits, school absenteeism due to illness and health related limitations were lower than in the total population, confirming that respiratory infections contribute to these health problems. In the children without respiratory infections, obesity was still associated with poorer health outcomes, but the associations were weaker than in the total population and, except for GP visits, no longer statistically significant. This suggests that respiratory infections do not explain all, but at least part of the health related problems found in excess in obese children.

### The role of parental perception of the child's body shape

Besides confounding by lifestyle factors, we assessed potential confounding by parental perception of the child's body shape. Ten percent of all parents (and 77% of the parents of obese children) found their child rather heavy or chubby and 23% of the parents found their child rather slim or slender. When the variable for perceived heaviness/chubbiness was added to the regression models, the associations with moderate overweight and obesity became stronger for each of the individual health outcomes except the RAND score. The adjusted odds ratios for the association with obesity increased to 6.41 (2.17; 18.9) for bronchitis, to 2.23 (1.26; 3.94) for use of antibiotics and by 0.1-0.2 for most of the other health outcomes. Parental perceived heaviness/chubbiness itself was not independently associated with any of the health outcomes, except the RAND score (adjusted β-0.83 (-1.40; -0.26)). When the variable for perceived slimness/slenderness was added to the regression models, this variable was independently and statistically significantly associated with a lower RAND score, with reported health-related limitations and with GP visits. Thinness itself was no longer independently associated with the RAND score in the model including parental perceived slimness/slenderness.

### Analyses in the complete cases and in the multiple imputed data

Tables 2 and 3 show the results of the analyses in the complete cases and the imputed dataset respectively. Comparison of these results shows some differences in the strengths of the associations. For 'absence from school due to illness' and for use of antibiotics the associations with obesity were statistically significant in the imputed data, but not in the complete cases, whereas the association between flu/serious cold and obesity was statistically significant in the complete cases but not in the imputed data. There was no consistent pattern of associations always being weaker or stronger in one analysis than in the other and all associations were in the same direction.

## Discussion

For the obese children our results suggest a consistent picture of poorer general health as compared to the normal weight children. The results indicate that this may be partly explained by a higher incidence of common childhood respiratory infections in obese children. The association between obesity and the use of antibiotics is also indicative for an association between obesity and infections. For the moderately overweight children, the results did not suggest poorer health. The analyses of the influence of parental perception of the child's body shape showed that parents who saw their child as slim/slender reported a lower RAND score, more GP visits and more health-related limitations than parents who did not see their child as slim/slender. Actually being thin was not associated with these outcomes in analyses adjusted for parental perception. When perceived heaviness/chubbiness was included in the regression models, the associations between obesity and the health outcomes became stronger rather than weaker (except for the RAND score), indicating that perception did not explain the associations that were observed between obesity and the health outcomes.

### Strengths and limitations of the study

Important strengths of the study were the large study population, the availability of measured, rather than reported, data on weight and height, the range of different health outcomes that could be studied, the possibility to adjust for prospectively collected indicators of parental health related behaviour and the possibility to assess the role of parental perception of the child's body shape.

A number of limitations have also to be considered.

Firstly, the health outcomes that were studied were based on questionnaire data and not on objective measurements. We therefore need to consider the potential influence of recall problems. We expect that parents will mostly have been able to recall the sort of events we asked about (such as "Did your child visit a GP during the last 2 months?"), but the possibility of recall errors can never be excluded. If parents' ability to recall health problems of the child was unrelated to the child's BMI status, incorrect recall will have caused 'noise' but not bias in our results. It could be hypothesized that parents with a relatively low 'health consciousness' or parents with parenting problems might have more obese children and might at the same time more frequently fail to recall disease episodes of their child. If this would be the case, this would have made the associations observed in our study weaker than the 'true' associations.

In addition to recall problems, we need to address the possibility that obese children are as healthy as normal weight children and that the only difference between them is the way their parents report on their children's health. The outcomes studied ranged from parameters that depend largely on subjective parental evaluation, such as the RAND score and health related limitations, to more objective measures like doctor diagnosed respiratory infections and use of antibiotics. Associations with obesity were observed for the more subjective as well as the more objective outcomes. However, although we asked about illnesses that were 'diagnosed by a doctor', a doctor's diagnosis depends not only on the (severity of the) actual illness, but also on parents' readiness to seek medical help. Indicators of parental health-related attitudes and behaviour, i.e. parental education, smoking and breast feeding were included in the analyses and did not substantially change the associations between overweight and the health outcomes. We therefore assume that the associations between obesity and parental reported health outcomes are not the result of confounding by parental health-related attitudes or behaviour. Also parental perception of the child's body shape, which was clearly involved in the association between thinness and general health, did not explain the associations between obesity and the health outcomes. We think therefore that it is unlikely that characteristics of the parents of obese children, rather than the actual health of obese children explain the associations we observed between obesity and the health outcomes studied.

Secondly, participation in the medical examination at the age of 8 years was lower than the response on the questionnaires, resulting in a relatively high proportion (44%) of missing data on measured weight and height. We followed common practice by conducting the analyses in the sub group of children with complete data. Because complete case analysis is liable to bias due to selective missing of data however, we repeated the analyses after missing data had been multiple imputed. The probability of data being missing was strongly associated with a number of variables measured in the study and we had parental reported weight and height data for most of the children who did not have their weight and height measured by the investigators. These two factors made us decide that multiple imputation was the best available method to deal with missing data in our study. Nevertheless, the relatively high percentage of missing values on BMI status remains a reason for cautious interpretation of the results both of the complete case analysis and the analysis in the imputed datasets. Comparison of the results of the analyses in the complete cases and in the imputed data showed some differences in the strengths of the associations. There was no consistent pattern of associations always being weaker or stronger in one analysis than in the other however and all associations were in the same direction. Both analyses suggested a consistent pattern of poorer health in the obese children.

Thirdly, the number of obese children in our study population was relatively low, especially in the complete case analysis, which may have limited the possibility to detect significant associations.

Finally, we specifically focused on health problems and diseases that have a high prevalence in children in the general population and we did therefore not address all the health problems obese children may face.

### Findings of other studies

Very few studies have investigated the association between overweight and the *general health outcomes *addressed in this paper. Pinhas-Hamiel et al. studied the health-related quality of life in Israeli overweight children and adolescents and observed that adverse associations were particularly strong in the physical domain as compared to the social, emotional and school domains [16]. In a study in Australian 4-5-year-old children, Wake et al investigated parent reported child global health, health related quality of life, mental health problems, asthma, sleep problems, injuries and special health care needs in relation to weight status [17]. They found no significant associations, except for a higher prevalence of special health care needs in obese children, and concluded that overweight and obese pre-school children experience few additional health burdens as compared to normal weight children. The authors suggest that morbidity rates may be higher in older children and the age difference between their study population and ours may, besides the differences in health outcomes studied, indeed explain the difference in the results between this study in pre-school children and our study. The same group recently published their results on comorbidities in overweight adolescents and reported that in this age group current obesity was associated with lower scores on 'physical health' and 'global health' [18].

With respect to the association between overweight and *respiratory infections*, evidence has been reported for a higher prevalence of wheezing, cough, bronchitis and pneumonia in U.S. children with a high BMI [19], for a higher incidence of respiratory and ear problems among Dutch overweight children [20] and for an association between obesity and the occurrence of otitis media with effusion in Korean children [21].

For adults, there is some evidence for associations between overweight and poor respiratory function [22], nose and throat complaints [23], common cold and influenza [24], respiratory diseases, including upper airways infections [25] and prescription of antibiotics and drugs for respiratory diseases [26,27]. Remarkably, the Centers for Disease Control recently published evidence that people who are obese but otherwise healthy may be at increased risk of severe complications and death from Influenza A (H1N1) [28].

### Interpretation of the results

Our data showed associations between obesity and respiratory infections and between obesity and the use of antibiotics. Similar results have been reported in the few studies that have covered these outcomes in children [19-21] and in adults [22-28]. Different explanations for these associations need to be considered. Reverse causation, illness causing overweight, cannot be excluded, but does not seem a plausible explanation. Illness tends to be associated with loss of appetite, and disease processes, especially infections with fever, increase metabolic rate [29], so that infectious disease is more likely to result in weight loss than in weight gain. Confounding could be an explanation. Although our analyses did not show evidence for confounding either by parental lifestyle or by parental perception of the child's body shape, we can not exclude the possibility that, for example, a low quality diet with a high energy and a low nutrient content could increase the risk of both overweight and respiratory infections. Given the limited effect on our results of breast feeding and smoking - factors that tend to be associated with families' eating habits - it seems unlikely however that an unhealthy diet could entirely explain the associations observed. Our results therefore suggest that obesity is likely to be an independent risk factor for doctor diagnosed respiratory infections in children.

How could obesity increase the risk of respiratory infections? Although we cannot tell from our data, we think that it is more likely that obesity may increase the severity and/or duration of respiratory infections than that it increases the incidence of infection. The observations on obese Influenza A (H1N1) patients seem to point in this direction. With respect to our data, this would imply that the obese children may not have had more infections than the normal weight children, but that they may have been ill longer or more seriously and therefore more often required GP attention and treatment with antibiotics. One possible explanation for an association between obesity and duration or severity of respiratory infections could be that excess body fat reduces lung volume, resulting in suboptimal ventilation and reduced clearance of micro-organisms from the airways. Another explanation could be that obesity influences immune responses to infection. Evidence for obesity related changes in immune responses has been observed in mice. Diet-induced obese mice, infected with influenza virus, were found to have a significantly higher mortality rate than their lean controls as well as elevated lung pathology [30]. Along with the increase in mortality, an altered immune response was observed in these mice, including delayed proinflammatory cytokine expression. Although these mechanisms seem plausible, the question if and to which extent they explain the associations between obesity and respiratory infections observed in epidemiological studies needs further study.

## Conclusion

Our results showed that obese children were more likely to be ill, to be absent from school due to illness, to experience health-related limitations and had a higher consumption of medical care than normal weight children. Our data suggest that childhood obesity is not only associated with risk of disease in adulthood, but that obese children may experience more illness and health related problems already in childhood. The high prevalence of the health outcomes studied imply a high burden of disease in terms of absolute numbers of sick children.

## Abbreviations

AOR: adjusted odds ratio; BMI: Body mass index; CI Confidence interval; GP: General Practitioner; MCAR: Missing completely at random; MICE: Multivariate imputation by chained equations; OR: Odds ratio; PIAMA: Prevention and Incidence of Asthma and Mite Allergy

## Competing interests

The authors declare that they have no competing interests.

## Authors' contributions

AHW and WJEB formulated the study question. AHW and SS analysed the data. AHW drafted the manuscript. MS gave statistical advice and performed the multiple imputation. HAS, BB, JCJ, MK, EAS and JG are principal investigators and senior researchers of the PIAMA study. They were responsible for the concept and the design of the PIAMA study and for data collection. All the authors were responsible for interpretation of the data and for critical revision of the manuscript and they all read and approved the final manuscript. The funding bodies had no influence on the content of the article or on the decision to submit it for publication.

## Pre-publication history

The pre-publication history for this paper can be accessed here:

http://www.biomedcentral.com/1471-2458/10/184/prepub
